# An examination of inpatient ward and secondary community care stay costs for individuals with complex mental health needs in the UK

**DOI:** 10.1371/journal.pmen.0000413

**Published:** 2025-09-08

**Authors:** Pooja Saini, Antony P. Martin, Jason C. McIntyre, Laura Sambrook, Anna Balmer, Hana Roks, Sam Burton, Peter Ashley-Mudie, Jackie Tait, Amrith Shetty, Rajan Nathan

**Affiliations:** 1 School of Psychology, Liverpool John Moores University, Liverpool, United Kingdom; 2 QC Medica, Liverpool, United Kingdom; 3 University of Chester and Cheshire and Wirral Partnership NHS Foundation Trust, Cheshire, United Kingdom; Bowen University, NIGERIA

## Abstract

Some people with mental health problems have such high levels of complex clinical and/or risk needs that those needs cannot be adequately met within generic mental health services. To design health and social provisions to better serve these people’s needs, it is necessary to first characterise the current provision. This study examines the cost element of this provision. This retrospective observational cohort study examined routinely collected healthcare service administrative data from a large UK-based NHS provider of community and hospital-based mental health services. Data were collected from medical records of individuals with complex mental health (CMH) needs aged ≥18 years old who had an inpatient ward stay between February 2000 until August 2021. Predictors of annual inpatient ward and secondary community care stay (residential/supported living/independent) costs were estimated using generalised linear models. Mean (median) annual total healthcare costs for 185 included adults were £106,847 (£109,651), comprising 16.4% from inpatient ward stay costs of £17,512 (£10,723) and 83.6% from secondary community care stay costs of £89,336 (£97,739). Associations varied across care context. Key predictors of inpatient stay cost included age, deprivation, and substance abuse. The primary diagnostic group of schizophrenia, schizotypal and delusional disorders (ICD10 codes: F20-F29) was found to be a predictor of greater secondary community care stay costs. Inpatient ward and secondary community care stay costs varied across patient characteristics. Additional research is warranted to further explore predictors identified in this study to prevent, promote, and monitor activities for individuals with differing CMH needs.

## Relevance statement

Some people with mental health problems have such high levels of complex clinical and/or risk needs that those needs cannot be adequately met within generic mental health services. To design health and social provisions to better serve these people’s needs, it is necessary to first characterise the current provision. This study examines the cost element of this provision. Individuals with complex mental health needs present a significant cost burden to the NHS. Additional research is warranted to further explore predictors identified in this study to prevent, promote, and monitor activities for individuals with differing CMH needs.

## 1 Introduction

### 1.1 Background/Rationale

In the UK, the National Health Service (NHS) providers of community and hospital-based mental health services are currently configured to provide largely community-based interventions. These services are supplemented by additional healthcare provision that is accessed on the basis of acuity/risk (i.e., inpatient services) or of diagnostic specificity [1]. Whilst there is a wide array of health services available for people with mental health problems, some people have such complex mental health (CMH) needs that the routinely available services are not best placed to address their needs [2]. The majority of people with CMH needs have a diagnosis of psychosis, treatment resistance, severe negative symptoms, and cognitive impairments [1,3]. It is also common that individuals with CMH needs live with coexisting mental health problems and physical health concerns resulting from poor lifestyle conditions and side effects of psychotropic medication [2].

Within this group of individuals with CMH needs there exists significant variation in support needs, which is reflected in the variation of provision. Inpatient hospitalisation stays are known to vary significantly between individuals with CMH needs and hospitalisations are well-known to present a significant cost to the NHS. As such, research is needed to better inform an evidence-based service delivery model for CMH service users. A delineation of the components of ‘complexity’ can help to provide an evidence base to support the development of appropriate services and support a move towards a ‘prevention’ approach in which the model of assessment and intervention at an earlier point may reduce the likelihood of the person becoming more ‘complex’ [1].

Studies have previously examined the direct costs to the healthcare system as well as the indirect costs to society associated with specialist mental healthcare [4–8]. Direct costs include: hospital services, pharmaceuticals, staff time, ambulances, and community care. Indirect costs include premature mortality, reduced health-related quality of life, reduced labour supply, lost output, lost tax revenue, transfer payments, and (unpaid) informal care provided by family or friends [9].

In a recent published report, poor mental health has been estimated to cost the UK economy £118 billion per year [10]. CMH needs, including schizophrenia and psychoses have been estimated at £11.8 billion in 2012 and for bipolar disorder, £5.2 billion in 2007 (equivalent to approximately £15.3 billion and £8.0 billion in 2022, respectively) [8].

Whilst the aforementioned studies provide detailed analyses of the costs associated with mental health problems (e.g., bipolar [4], schizophrenia [5], serious mental illness excluding personality disorder [8]), no previous study has focused specifically on serious and complex mental health problems collectively, i.e., those whose needs are so complex that they require more specialist input than is offered by routinely available community and inpatient services. Additionally, previous research has lacked the inclusion of people with personality disorders, perhaps due to the complexity of diagnosing this mental health condition. Focusing on this group with CMH, and understanding which patient profiles are likely to incur most inpatient ward and secondary community care stay costs, may help policymakers and service providers better allocate health resources and plan health budgets across care pathways. The focus of our study is to gain an in-depth understanding of a cohort of service users with CMH needs and to provide an exploration of potential predictors of increased inpatient ward stay and secondary community care stays and associated costs.

## 2 Materials and methods

### 2.1 Study population, data sources, and CMH categorisation

This retrospective observational cohort study used routinely collected healthcare service administrative data from inpatient admissions in a large UK-based NHS provider of community and hospital-based mental health services based in the Northwest area of England. Cheshire and Wirral Partnership NHS Foundation Trust (CWP) provides a wide range of community and inpatient, physical, all-age disability and mental health care services. The services extend to other areas in the Northwest including, Liverpool, Warrington and Halton. CWP services a catchment area of approximately 2.4 million people with a diverse population, including areas of high deprivation. CWP’s provision includes the delivery of care to a specific cohort of patients defined as having ‘complex mental health needs.’

Complex mental health (CMH) needs is a broad term used to describe patients who have received a health funded package commissioned by Wirral National Health Service Clinical Commissioning Group (NHS CCG) either in an inpatient or a community setting. This study sample comprised service users with CMH needs who were at the time of the study in an inpatient setting, or in a community setting and subject to Section 117 aftercare. Data were collected from the CWP electronic health records (Care Notes) for individuals with CMH aged ≥18 years old from February 2000 until August 2021. Data received were pseudonymised and assigned a unique identification code. Following a consultation process with the wider stakeholder team, the final data set was completed between November 2021 and April 2022 by data analysts based at the NHS Trust.

### 2.2 Measures

Baseline characteristics were summarised for individuals with CMH, which included: age, gender, ethnicity, marital status, red flag for alcohol, red flag for smoker, red flag for substance abuse, and ICD10 primary diagnosis. The term ‘red flag’ flags for those with addictions due to signs and symptoms which indicate the presence of an addiction. In addition, first half of postcode of patient residence were mapped onto Middle Layer Super Output Area (MSOA) which were then subsequently linked to an Index of Multiple Deprivation (IMD) quintile [11]. From the 2019 census, there were 7,201 MSOAs in England (6,791) and Wales (410) used in the 2015 Indices of Deprivation in England [11] MSOAs have a minimum size of 5,000 residents and 2,000 households with an average population size of 7,800. They fit within local authority boundaries. The MSOAs were mapped onto IMD quintiles, which combine seven indicators (income, employment, health deprivation and disability, education, skills and training, barriers to housing and services, crime, and living environment) into a single-deprivation index [11]. Data for patients registered at a permanent address outside of England were excluded from the deprivation analysis as information could be not applied for the same index.

### 2.3 Inpatient ward and secondary community care stay costs

Average yearly inpatient ward and secondary community care stay cost per patient depends on the frequency and duration of the admission. Information on inpatient ward and secondary community care stays were collected so that frequency and duration could be estimated in days. For each patient, the proportion of year as an inpatient stay or secondary community care stay was then calculated for each patient (this was standardised considering a year). Annual costs have previously been estimated based on an analysis conducted by Care Quality Commission (CQC) in 2018. The median daily costs of an inpatient mental health ward bed and secondary community care bed were £354 and £339, respectively. As such, an inpatient ward bed has an estimated yearly cost of £129,299 and a secondary, community care bed has an estimated yearly cost of £123,820 (assuming full occupancy) [12].

### 2.4 Statistical methods

Descriptive analyses were conducted to produce a profile of the patient group. The costs in this analysis refer to the cost per day of a bed by rehabilitation ward type and used “all ward types” and “community”. For each patient, inpatient ward and secondary community care bed day costs were aggregated into annual costs by year. To investigate patterns of inpatient ward and secondary community care stay costs across patient characteristics, we examined the distribution of costs and conducted multivariate regression analyses. Explanatory variables were based on factors associated with utilisation of healthcare in prior research [8,13].

We used a generalised linear model (GLM) with individual random effects, which allows for the non-normal distribution of healthcare costs and models the mean directly, avoiding the need to transform the data. The choices of model were tested for multicollinearity [14] and standardized residuals were explored with a Q-Q plot [15]. The model was based on a Poisson distribution. All analyses were conducted in R (version 4.1.2) and R Studio (version 2022.07.1) [16]. This study is reported as per the Strengthening the Reporting of Observational Studies in Epidemiology (STROBE) guidelines [17].

### 2.5 Ethics

Ethical approval was obtained from the NHS Health Research Authority and West Midlands - Coventry & Warwickshire Research Ethics Committee: [REC Ref: 21/WM/0020] Integrated Research Application System (IRAS) prior to study commencement. Ethical approval was received on 19th March 2021 from HRA and Health and Care Research Wales (HCRW).

## 3 Results

### 3.1 Sample

The study sample included 185 adults with CMH needs who had an inpatient ward and secondary community care stay in CWP between February 2000 and August 2021. Approximately three-quarters of the sample (74.1%) were observed for six years or more.

The first data column of Table 1 shows the distribution of characteristics across the sample. The mean (median) age was 44.8 (SD 13.4) (45 [IQR: 33–55]) years, the majority (91.9%) were white, the majority (82.4%) were single and were more likely to have permanent residence in a more deprived area than the general population. In the sample, 37.3% were red flagged for smoking, 12.4% were red flagged for substance use, and 9.7% were red flagged for alcohol use.

**Table 1:** Patient characteristics and mean annual healthcare costs.

Index of multiple deprivation quintile							
1 (most deprived)	63 (38.0)	12,401	15,245	83,213	40,616	95,614	46,346
2	6 (3.6)	8901	9376	103,781	10,762	112,682	18,304
3	76 (45.8)	18,509	21,691	89,430	40,028	107,939	48,126
4	10 (6.0)	39,321	25,924	83,591	24,739	122,911	40,271
5 (least deprived)	11 (6.6)	9110	7292	97,281	40,103	106,392	44,171
Red Flag Smoking							
No	116 (62.7)	15,293	20,350	95,697	34,395	100,844	48,665
Yes	69 (37.3)	21,242	19,978	85,551	41,070	116,939	41,203
Red Flag Substance Abuse							
No	162 (87.6)	15,850	18,798	87,867	38,960	103,717	44,760
Yes	23 (12.4)	29,214	26,807	99,678	37,953	128,892	53,804
Red Flag Alcohol Abuse							
No	167 (90.3)	16,890	20,694	89021	39,508	105,911	47,931
Yes	18 (9.7)	23,275	16,355	92,254	33,957	115,529	30,813
ICD10 code							
Schizophrenia, schizotypal and delusional disorders (F20-F29)	115 (63.5)	17,050	20,870	95,975	36,426	113,026	43,737
Mood affective disorders (F30-F39)	22 (12.2)	11,728	11,560	73,981	36,360	85,710	39,031
Disorders of adult personality and behaviour (F60-69)	12 (6.6)	18,784	20,966	74,730	41,477	93,515	43,008
Mental and behavioural disorders due to psychoactive substance use (F10-19)	9 (5.0)	17,042	25,985	55,413	41,991	72,455	64,653
Neurotic, stress-related and somatoform disorders (F40-F48)	7 (3.9)	16,434	6418	82461	42,199	98,895	43,984
Behavioural syndromes associated with psychological disturbances (F50-59)	5 (2.8)	28,892	14,034	132,742	23,697	161,634	31,390
Disorders of psychological development (F80-89)	4 (2.2)	22,641	25,131	104,900	5144	127,541	21,677
Mental retardation (F70-79)	3 (1.7)	36,937	34,050	74,476	36,592	111,413	70,637
Other	4 (2.2)	45,291	21,327	66,947	41,498	112,238	62,620
Time since first diagnosis (years)							
0-1	4 (2.5)	36,770	14,907	77,988	39,562	114,758	43,450
2-5	37 (23.4)	27,555	25,680	68,204	46,398	95,759	55,610
6+	117 (74.1)	36,770	24,265	77,988	43,400	114,758	50,758

*ICD10* International Classification of Diseases 10^th^ Revision, *N* number, *SD* standard deviation.

A total of 115 (63.5%) individuals received a primary diagnosis of ‘schizophrenia, schizotypal and delusional disorders (F20-F29).’ The second and third most common primary diagnoses were ‘mood affective disorders (F30-F39)’ and ‘disorders of adult personality and behaviour (F60-69)’ with 22 (12.2%) and 12 (6.6%) individuals diagnosed, respectively.

**Fig 1:**
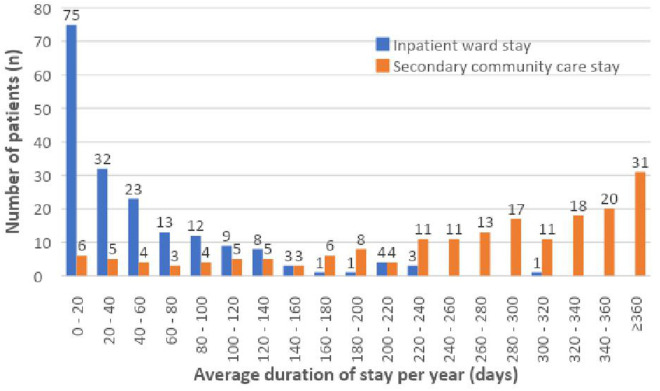
Average duration of inpatient ward and secondary community care stays per year.

Fig 1 illustrates the average length of inpatient ward and secondary community care stay per year. A mean (median) stay of 49.5 (SD 58.1) (30.3 [IQR: 16.4-81.3]) days was identified. However, 16.2% (n = 30) of patients experienced an average length of inpatient ward stay per year more than 100 days. Secondary community care stay was identified with a mean (median) stay of 263.5 (SD 114.8) (288.3 [IQR: 198.7-342.5]) days. Indeed, 43.2% (n = 80) of patients experienced an average length of secondary community care stay more than 300 days.

As shown in Table 1, mean (median) annual per patient inpatient ward stay costs were £17,512 (£10,723), secondary community care stay costs were £89,336 (£97,739), and total costs were £106,847 (£109,651). Total costs were made up of 16.4% from inpatient ward stay and 83.6% from secondary community care stays, respectively. Mean inpatient ward stay costs were lower with increased age and higher with white ethnicity, female gender, and IMD quintile 4 (Q4, less deprived). Mean inpatient ward stay and secondary community care stay costs were also higher with red flags for smoking, substance use, and alcohol use. Patients with the most common primary diagnosis of ‘schizophrenia, schizotypal and delusional disorders (F20-F29)’ had a mean annual inpatient ward stay cost of £17,050 and mean secondary community care stay costs of £95,975. The second and third most common primary diagnoses were ‘mood affective disorders (F30-F39)’ and ‘disorders of adult personality and behaviour (F60-69)’ with a mean annual inpatient ward stay cost of £11,728 and £18,784, respectively, and mean secondary community care stay costs of £73,981 and £74,730, respectively.

Mean total costs were lower by age and higher with white ethnicity, female gender, and IMD quintile 4 (Q4, less deprived). Mean total costs were also higher with red flags for smoking, substance use, and alcohol use. Patients with the most common primary diagnosis of ‘schizophrenia, schizotypal and delusional disorders (F20-F29)’ had a mean annual total cost of £113,026, followed by ‘mood affective disorders (F30-F39)’ and ‘disorders of adult personality and behaviour (F60-69)’ with a mean annual total cost of £85,710 and £93,515, respectively.

### 3.2 Association of characteristics with costs

The association of each patient characteristic with the difference in estimated yearly inpatient ward stay, secondary community care stay and total stay costs (marginal effects from random effects GLM) is presented in Table 2. After adjusting for other observable characteristics for estimated yearly inpatient ward stay, higher costs were identified for patients who were younger (*p* = 0.037) (although this was only evidenced in the 60 + age group compared to the 18–40 age group), a permanent resident of IMD quintile 3 (Q3) (median) (p = 0.017) and Q4 (less deprived) (*p* = 0.016) and had a red flag for substance use (*p = *0.075). After adjusting for other observable characteristics for estimated yearly secondary community care stay, patients who received a primary diagnosis of ‘schizophrenia, schizotypal and delusional disorders (F20-F29)’ had a higher mean annual cost of £22,217 (*p* = 0.002). Likewise, after adjusting for other observable characteristics for estimated total costs, patients who received a primary diagnosis of ‘schizophrenia, schizotypal and delusional disorders (F20-F29)’ had a higher mean annual cost of £23,622 (p = 0.007). Longer duration of time since primary diagnosis was suggestive of lower average yearly total costs but this variable was not significant.

**Table 2:** Association of patient and practice characteristics (average marginal effects, £ per year).

	Inpatient ward stay cost (£)			Secondary community care stay (£)			Total cost (£)		
Variable	AME	95% CI	*p* value	AME	95% CI	*p* value	AME	95% CI	*p* value
Age									
18 to <40	Ref.			Ref.			Ref.		
40 to <60	2996.64	(-4155.48, 10,148.76)	0.412	-9053.23	(-24,042.49, 59,36.04)	0.237	-7284.21	(-25418.12, 10849.69)	0.431
60+	-8664.86*	(-16,793.52, -536.18)	0.037	-4638.11	(-27,067.37, 17,791.14)	0.685	-13734.92	(-39903.28, 12433.43)	0.303
Sex									
Female	Ref.			Ref.			Ref.		
Male	-2752.11	(-9312.34, 3808.11)	0.411	-2676.39	(-16,296.23, 10,943.45)	0.700	-5499.85	(-22079.65, 11079.93)	0.515
Ethnicity									
Black, minority ethnicities	Ref.			Ref.			Ref.		
White	3154.11	(-7985.93, 14,294.15)	0.579	-1207.47	(-26,576.77, 24,161.82)	0.926	2443.76	(-27787.12, 32674.65)	0.874
Marital status									
Other	Ref.			Ref.			Ref.		
Single	549.15	(-9587.71, 10,686.01)	0.915	-2325.69	(-21,977.82, 17,326.44)	0.817	-1992.43	(-26139.01, 22154.14)	0.871
Index of multiple deprivation quintile									
1 (most deprived)	Ref.			Ref.			Ref.		
2	1281.82	(-16,388.05, 18,951.71)	0.887	40632.22	(-10,185.87, 91,450.3)	0.117	40799.39	(-17776.51, 99375.29)	0.172
3	7625.45*	(1368.85, 13,882.04)	0.017	3306.29	(-10,545.36, 17,157.94)	0.640	10388.56	(-6274.71, 27051.84)	0.221
4	19,484.71*	(3692.15, 35,277.26)	0.016	246.48	(-27,825.6, 28,318.56)	0.986	23201.83	(-12706.7, 59110.36)	0.205
5 (least deprived)	-4920.22	(-13,396.85, 3556.41)	0.255	15778.92	(-11,111.86, 42,669.69)	0.250	10994.87	(-19908.81, 41898.56)	0.485
Red Flag Smoke									
No	Ref.			Ref.			Ref.		
Yes	1383.92	(-5555.22, 8323.07)	0.696	224.37	(-14,655, 15,103.74)	0.976	1946.53	(-16063.5, 19956.57)	0.832
Red Flag Substance									
No	Ref.			Ref.			Ref.		
Yes	13,308.54^+^	(-1327.9, 27,944.97)	0.075	6770.27	(-17,424.12, 30,964.67)	0.583	18132.73	(-12348.11, 48613.59)	0.243
Red Flag Alcohol									
No	Ref.			Ref.			Ref.		
Yes	-5707.74	(-14,884.91, 3469.44)	0.223	-1828.53	(-26,409.22, 22,752.16)	0.884	-7512.78	(-36023.39, 20997.83)	0.605
ICD10 code									
Schizophrenia, schizotypal and delusional disorders (F20-F29)	Ref.			Ref.			Ref.		
Other	-36.35	(-6935.33, 6862.62)	0.992	-22217.26	(-36,564.87, -7869.64)	0.002**	-23622.07**	(-41012.71, -6231.42)	0.007
Time since first diagnosis (years)									
0-1	Ref.			Ref.			Ref.		
2-5	-7008.33	(-32,203.51, 18,186.86)	0.586	-23921.18	(-64,934.58, 17,092.22)	0.253	-35437.77	(-89006.48, 18130.94)	0.194
6+	-19,176.22	(-42,570.07, 4217.62)	0.108	10597.26	(-29,206.16, 50,400.69)	0.602	-11070.29	(-62725.61, 40585.02)	0.674

Marginal effects from GLM with Poisson distribution and log link with individual random effects.

*CI* confidence interval, *GLM* generalised linear model, *ICD10* International Classification of Diseases 10^th^ Revision.

+ = < 0.10, * = < 0.05, ** = < 0.01, *** = < 0.001.

## 4 Discussion

People with severe mental illness experience significant reductions in life expectancy compared with the general population; with 14.5 years loss in men with schizophrenia-spectrum and bipolar disorders and 13.2 years in women [18]. Evidence-based interventions to tackle this mortality gap and consideration of how to adequately manage scarce healthcare resources to promote allocative and technical efficiency are needed. Previous research has examined the direct costs to the healthcare system as well as the indirect costs to society associated with specialist mental healthcare [4–8]. Although studies have focused on costs for mental health [10] there has been no research to our knowledge for costs associated with service utilisation for those with CMH needs. This study has added to the limited research of this nature to further understand predictors for people with CMH need and provides meaningful data per patient cost.

Estimates of inpatient ward and secondary community care stay costs for individuals with CMH needs are required to inform resource allocation and for service planning. Moreover, an in-depth understanding of variation within individuals defined as complex provides opportunity to identify and subsequently address unmet need and inefficiencies which exist. This study highlights the significant cost burden to the NHS associated with the management of individuals with CMH needs. Our research examined sociodemographic and clinical information to inform predictors of inpatient ward and secondary community care stay costs for individuals with CMH for a large UK-based NHS provider of community and hospital-based mental health services. We found that inpatient ward stay costs were higher for patients who were younger, had a permanent resident of in an IMD Q3 and Q4 area, and had a red flag for substance use. This suggest that resources should be targeted at younger people with CMH needs, particularly those living in areas of high deprivation and those with substance abuse problems. However, these results need to be interpreted cautiously as this is a large population and without clear evidence to support specific interventions, it is impossible to know if further investment in these groups will be cost-effective.

The age disparity in costs can be explained as individuals with CMH needs become older their associated inpatient ward stay costs reduce (with less frequent and lower duration admissions) as their treating clinicians and the individuals themselves learn how to better manage their symptoms and associated complications. Indeed, older individuals with CMH needs are more likely to have spent more time in contact with health services than younger individuals. As such, expected average costs yearly costs can be expected to be lower.

The finding of increased costs associated with individuals who were permanent residents of neighbourhoods in IMD Q3 and Q4 should be interpreted with caution. An important limitation relates to the insensitivity of IMD matched to each MSOA as within each MSOA there may be signiﬁcant variation in terms of deprivation [11]. Similarly, other research has found that people with severe mental illness are more likely to live in deprived communities and as such we recommend that our findings should be investigated further [19,20]. Future research should also include lower super output area (LSOA) data to better inform local variation associated with deprivation.

The finding for increased costs associated with a ‘red flag for substance use’ and inpatient ward stay was anticipated. Previous research has also found that the presence of substance use disorders (across a broad spectrum of substance types) in individuals with CMH needs was associated with an increased risk of psychiatric admissions, psychiatric emergency department presentations and longer in-patient stays [21]. Comorbid substance use disorders are known to be more prevalent in CMH populations compared with the general population [22].

Although flags for alcohol abuse and smoking were not found to be significant, although rates were higher or similar to the general population, both have previously been identified as risk factors for physical health complications [23]. Previous systematic reviews and meta-analyses have found that lifetime alcohol use disorders are known to more frequently affect people with schizophrenia [24] and people with bipolar disorders [25,26]. A recent study found the prevalence of alcohol misuse in individuals with severe mental illness to be higher (13.5% versus 3.7%) [23]. Likewise, the prevalence of smoking in individuals with severe mental illness has also been found to be higher compared with the general population (46.1% versus 27.7%) [23]. Another important consideration is that individuals with CMH needs are more likely to engage in polysubstance use [22]. As such, additional research should examine these flags further and consider system-wide economic consequences associated with increased alcohol abuse and smoking.

We also found that a primary diagnosis of ‘Schizophrenia, schizotypal and delusional disorders (F20-F29)’ was associated with higher secondary community care costs compared with other primary diagnoses. Although we found that a primary diagnosis of ‘Schizophrenia, schizotypal and delusional disorders (F20-F29)’ was associated with increased secondary community care costs. The National Institute for Health and Care Excellence (NICE) quality standard [QS80] on psychosis and schizophrenia states that adults with a first episode of psychosis should start treatment in early intervention in psychosis services within two weeks of referral. Previous research has found that the sooner individuals are able to access evidence-based treatments after the onset of psychosis, the better the outcomes they achieve [27].

Given that the focus of the study was on those services users with particularly complex needs (to the extent that their needs could not be met within generic mental health services), the sample size was small when compared with the large catchment population. Additionally, this study was limited by a high level of missing data relating to several demographic and physical health variables not included in this analysis, such as religious status, body mass index (BMI) and blood pressure. We also note that there was some skewing for inpatient ward and community care stay. As such, it is important to interpret our cost estimates with caution. We highlight the need for a national joined dataset for such research. As the original dataset was not designed for research, some core information was not routinely collected. Data were assumed missing not at random and therefore multiple imputation methods were precluded. Further, the management of individuals with CMH needs who received secondary community care outside of the NHS were not included in this analysis.

Additionally, recorded data might be subject to recording errors or adjudication errors, and further, the reliability of the coding of CMH need data might have changed over time. However, there was no evidence of changes in treatment coding during the study. Despite these limitations, we believe the study and its ﬁndings provide useful insights and guidance for future studies as more data becomes available.

Further research is warranted to explore CMH need subgroups to better inform the provision of appropriate and timely health prevention, promotion, and monitoring activities. A larger study with multiple sites and increased sample size is recommended to further explore variation in clinical and economic outcomes associated with individuals living with CMH needs.

## 5 Conclusions

Inpatient ward and secondary community care costs for individuals with mental health needs vary across clinical and socioeconomic characteristics; however, further variation exists within individuals defined as complex. This research highlights the significant cost burden to the NHS and variation associated with the management of individuals with CMH needs. Observable patient characteristics in medical records merit further exploration in larger multi-site studies to better inform resource allocation and improve targeting of evidence-based interventions.
